# Commissioning of total body irradiation using plastic bead bags

**DOI:** 10.1093/jrr/rraa072

**Published:** 2020-09-02

**Authors:** Yuichi Akino, Shintaro Maruoka, Katsuyuki Yano, Hiroshi Abe, Fumiaki Isohashi, Yuji Seo, Keisuke Tamari, Takero Hirata, Manabu Kawakami, Yoshiki Nakae, Yoshihiro Tanaka, Kazuhiko Ogawa

**Affiliations:** Oncology Center, Osaka University Hospital, Suita, Osaka 565-0871, Japan; Nippon Life Hospital, Nishi-ku, Osaka 550-0006, Japan; Nippon Life Hospital, Nishi-ku, Osaka 550-0006, Japan; Nippon Life Hospital, Nishi-ku, Osaka 550-0006, Japan; Nippon Life Hospital, Nishi-ku, Osaka 550-0006, Japan; Department of Radiation Oncology, Osaka University Graduate School of Medicine, Suita, Osaka 565-0871, Japan; Department of Radiation Oncology, Osaka University Graduate School of Medicine, Suita, Osaka 565-0871, Japan; Department of Radiation Oncology, Osaka University Graduate School of Medicine, Suita, Osaka 565-0871, Japan; Department of Radiation Oncology, Osaka University Graduate School of Medicine, Suita, Osaka 565-0871, Japan; Nippon Life Hospital, Nishi-ku, Osaka 550-0006, Japan; Nippon Life Hospital, Nishi-ku, Osaka 550-0006, Japan; Department of Radiation Therapy, Japanese Red Cross Society Kyoto Daiichi Hospital, Kyoto 605-0981, Japan; Department of Radiation Oncology, Osaka University Graduate School of Medicine, Suita, Osaka 565-0871, Japan

**Keywords:** total body irradiation, *in vivo* dosimetry, treatment planning, Acuros XB

## Abstract

The goal of total body irradiation (TBI) is to deliver a dose to the whole body with uniformity within ±10%. The purpose of this study was to establish the technique of TBI using plastic bead bags. A lifting TBI bed, Model ORP-TBI-MN, was used. The space between the patient’s body and the acrylic walls of the bed was filled with polyacetal bead bags. Patients were irradiated by a 10 MV photon beam with a source to mid-plane distance of 400 cm. The monitor unit (MU) was calculated by dose-per-MU, tissue-phantom-ratio and a spoiler factor measured in solid water using an ionization chamber. The phantom-scatter correction factor, off-center ratio and the effective density of the beads were also measured. Diode detectors were used for *in vivo* dosimetry (IVD). The effective density of the beads was 0.90 ± 0.09. The point doses calculated in an I’mRT phantom with and without heterogeneity material showed good agreement, with measurements within 3%. An end-to-end test was performed using a RANDO phantom. The mean ± SD (range) of the differences between the calculated and IVD-measured mid-plane doses was 1.1 ± 4.8% (−5.9 to 5.0%). The differences between the IVD-measured doses and the doses calculated with Acuros XB of the Eclipse treatment planning system (TPS) were within 5%. For two patients treated with this method, the differences between the calculated and IVD-measured doses were within ±6% when excluding the chest region. We have established the technique of TBI using plastic bead bags. The TPS may be useful to roughly estimate patient dose.

## INTRODUCTION

Total body irradiation (TBI) has been used as the conditioning regimen prior to allogeneic hematopoietic stem cell transplantation (HSCT) for treatments of various hematological diseases including leukemia, malignant lymphoma, myelodysplastic syndrome and severe aplastic anemia [1–3]. For myeloablative conditioning, a prescribed dose ranging from 12 to 13.2 Gy has been commonly used to attenuate the immunity of the recipient and to destroy cancer cells [4]. For reduced-intensity stem cell transplantation (RIST) combined with TBI, a prescribed dose ranging from 2 to 6 Gy has been used [5, 6]. To irradiate the whole patient body, various techniques, such as long source-to-surface distance (SSD), moving bed [7, 8] and helical tomotherapy [9, 10], have been used. For the long SSD technique, the beam-on time is very long because of the low dose rate. Compared to anterior-to-posterior (AP) and posterior-to-anterior (PA) irradiation, bilateral irradiation in the supine position is more comfortable for patients. However, irregular patient body surface orthogonal to the incident beams leads to inhomogeneous delivered dose. Generally the goal of beam delivery in TBI is to achieve ±10% uniformity in the whole body [11, 12]. However, variations in the patient thickness and heterogeneity in lungs result in over- or under-dosage. To improve the dose uniformity, various techniques have been developed, such as use of compensator [13, 14], field-in-field [15], intensity-modulated radiotherapy [16] and adjustments of the speed of the moving couch [17]. Recently, total marrow irradiation (TMI) using helical tomotherapy has also been performed to reduce toxicity [9, 18, 19]. If patients are laid in a water-equivalent material, the physical thickness is the same at all anatomical sites, although heterogeneities inside the patient body affect the effective depth. This technique is simple and patients can be treated with the same monitor units (MUs) if the prescribed dose is the same. The purpose of this study was to establish the technique of TBI using bags filled with plastic beads. We have provided formulas to calculate the point dose at the mid-plane of the patient’s head, neck, chest and umbilicus levels with consideration of the additional compensator, air gap around the patient’s head and heterogeneity in the lungs. For TBI, *in vivo* dosimetry (IVD) is useful to confirm the validity of treatments and to minimize treatment errors [17, 20–22]. We also evaluated the irradiated dose to patients during treatments using IVD detectors. The measured doses were compared with the calculations. The calculation accuracy of a treatment planning system (TPS) for the long-SSD method was also evaluated.

**Fig. 1.**
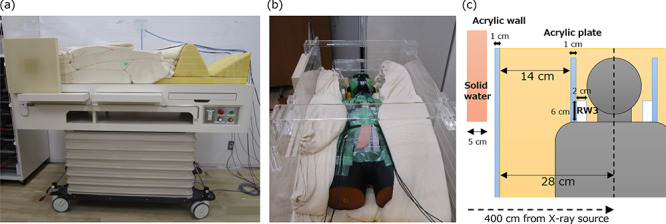
(**a**) A photograph of the TBI bed with plastic beads and compensators. (**b**) A photograph of the Rando phantom positioned on the TBI bed. The gaps between the phantom and the acrylic walls are filled with the bead bags. Cables of the IVD2 detectors are also shown. (**c**) Geometry of the bed and compensator components.

## MATERIALS AND METHODS

### Instruments and patient setup

A lifting TBI bed, Model ORP-TBI-MN (Orion Electric Co., Ltd., Nagoya, Japan), was used. Photographs and the geometry of the bed are shown in Fig. 1. This bed consisted of a wooden tabletop with acrylic walls with a thickness of 1 cm. The distance between the two acrylic walls was 56 cm. The space between the patient’s body and the walls was filled with plastic beads encapsulated into fabric bags. The beads were polyacetal with a density of 1.41 g/cm^3^. There were four types of bag sizes: 1-, 2-, 3- and 5-kg weights. Patients were laid on the table top in the supine position. For RIST treatment, a bead bag was placed under each of the patient’s upper arms to reduce the lung dose by overlapping the patient’s arms and lungs. Additionally two acrylic plates were placed beside the patient’s head for comfort. For the head and neck region, the gaps between the patient’s body and the acrylic plates were not filled with the bead bags. To compensate for the airgap around the patient’s head, a solid water phantom (Tough Water, Kyoto Kagaku, Kyoto, Japan) 5 cm-thick was placed upstream of the acrylic wall to cover the patient’s head and neck. Because a patient’s neck is thinner than the head, the RW3 inserts of the I’mRT phantom (IBA Dosimetry, Schwarzenbruck, Germany) were attached to the acrylic plates to cover the patient’s neck. The size of the inserts was 2 × 6 × 16 cm^3^.

A TrueBeam linear accelerator (Varian Medical Systems, Paro Alto, CA, USA) was used for irradiation. The photon energy, gantry angle, collimator angle and nominal field size at the isocenter were 10 MV, 270°, 45° and 40 × 40 cm^2^, respectively. The distance between the source and the mid-plane of the bed was 400 cm. The SSD to the surface of the acrylic wall was 371 cm. The dose rate for treatment was 200 MU/min. The beam output linearity and the dose rate stability were evaluated using a Farmer-type ionization chamber (model 30013, PTW Freiburg, Freiburg, Germany) and were within ±1%.

### Calculation of MUs

The MUs for treatment was calculated using following formula:

(1) \begin{equation*} \mathrm{MU}=\frac{D_{\mathrm{presc}}}{\mathrm{DMU}\bullet \mathrm{TPR}\bullet \mathrm{SF}} \end{equation*}

where *D*_presc_, DMU, TPR and SF represent the prescribed dose, dose-per-MU, tissue-phantom-ratio and spoiler factor, respectively. To calculate the DMU, the dose was measured with a Farmer-type ionization chamber at 10 cm depth in solid water (Supplementary Fig. 1 a, see online supplementary material). The size of the solid water was 40 × 40 cm^2^. The source-to-detector distance (SDD) was 400 cm. The ion recombination correction factor (*k*_s_) and polarity correction factor (*k*_pol_) were measured using the Japan Society of Medical Physics (JSMP) standard dosimetry protocol (JSMP 12) [23]. The TPR was measured at a SDD of 400 cm by increasing the depth from 10 to 28 cm (Supplementary Fig. 1b). In this study, the absorption of the acrylic wall was considered as the SF and calculated as the ratio of the detector readings with and without the wall.

### Point dose calculation

The point doses (*D*) at the mid-plane of patient’s head, neck, chest and umbilicus were calculated using following formula:

(2) \begin{equation*} D=\mathrm{MU}\bullet \mathrm{DMU}\bullet \mathrm{TPR}\bullet \mathrm{SF}\bullet \mathrm{PSCF}\bullet{\mathrm{G}}_{\left(d,d0\right)}\bullet \mathrm{OCR} \end{equation*}

where the PSCF and OCR represent the phantom scatter correction factor and the off-center ratio, respectively. The PSCF was measured at 20 cm depth (the middle of the solid water) with decreasing the height from 40 to 16 cm (Supplementary Fig. 2a, see online supplementary material). Because the height of the solid water was 40 cm for measurement of the DMU, the reference height for PSCF was 40 cm. The *G_(d,d0)_* represents the correction factor for an inverse square law:

(3) \begin{equation*} {G}_{\left(d,d0\right)}={\left(\frac{\mathrm{SSD}+d0}{\mathrm{SSD}+d}\right)}^2 \end{equation*}

where SSD represents the source-to-surface distance at the acrylic wall of the bed. This value is unity when calculating for the depth at mid-plane (*d*0). The OCR was defined as the beam profile in the horizontal direction, and the axis was parallel to the longitudinal direction of the bed. The OCR was measured using the Farmer-type ionization chamber at 28 cm depth in solid water. The measurements were repeated by moving the solid water horizontally on the bed (Supplementary Fig. 2b).

### Relative density of plastic beads

To investigate the effective density of the plastic beads, the dose was measured at 28 cm depth in the solid water with the acrylic wall. The SDD was 400 cm. Then the measurements were repeated by replacing part of the solid water by plastic bead bags (Supplementary Fig. 3, see online supplementary material). The water-equivalent-depth (WED) for the combination of the plastic beads and the solid water (WED_solid water + beads_) was calculated using a depth–TPR approximation curve generated from the measured TPR. The density of the plastic beads relative to water was calculated as follows:

(4) \begin{equation*} {\rho}_{\mathrm{beads}}=\frac{\mathrm{WED}-{d}_{\mathrm{solid}}}{d_{\mathrm{beads}}} \end{equation*}

where *d*_solid_ and *d*_beads_ represent the physical thickness of the solid water and bead bags, respectively.

### Phantom measurements

To investigate the accuracy of the dose calculation with equation (2), the I’mRT phantom was placed at the center of the bed, and the dose was measured using the Farmer-type ionization chamber (Supplementary Fig. 4, see online supplementary material). The plastic bead bags filled both sides of the gaps between the phantom and the acrylic walls. The measurements were performed at the mid-plane of the phantom (i), and the position was shifted by 3 cm in the distal direction (ii) to evaluate the validity of the inverse square law. To evaluate the calculation accuracy for inhomogeneous material, half of the RW3 plates (8 cm thickness) were replaced by lung-equivalent plates (Tough Lung: Kyoto Kagaku) and measurement was performed (iii). For Tough Lung, the WED was calculated by multiplying the physical thickness by 0.3.

### *In vivo* dosimetry

To evaluate the patient dose during the treatment, IVD^TM^2 and QED^TM^ diode detectors for 6–12 MV photon energies (Sun Nuclear Corp., Melbourne, FL, USA) were used. Although the sensitive volume of the diode is located at 5 mm from the surface, the detector contains a 1.9 g/cm^2^ brass shield at one side. Therefore, the detectors have 1.4 cm of additional buildup. Each detector was calibrated for both brass-shielding and for the opposite side, because the detectors located at proximal and distal sides of patients were irradiated from the brass-shielded and the opposite sides, respectively. When the bed is rotated to irradiate patients from the opposite side, the IVD detectors remain, resulting in irradiation to the detectors from both sides. For calibration of the QED, the detectors were placed at 10 cm depth in solid water and irradiated by 10 MV photon beams at an SDD of 400 cm. The dose at the detector position was determined by measurements using the Farmer-type ionization chamber at 11.5 and 10 cm depths in the solid water for brass-shielded and opposite sides, respectively. For the shielded side, we could not measure the dose at 11.4 cm depth because of the limitation of the solid water we used, although the influence would be negligibly small.

Before treatments, the IVD detectors were attached to both proximal and distal sides of the body at patient head, neck, chest and umbilicus levels. At the chest level, the detectors were placed between the patient’s body and arms. The point dose (*D*_x_) at physical depth *d*_x_ was calculated from the doses measured with the proximal and distal detectors using the following formula:

(5) \begin{equation*} {D}_x={D}_{\mathrm{IVD}}\bullet \frac{{\mathrm{PDD}}_{\left({\mathrm{WED}}_x\right)}\bullet{\mathrm{G}}_{\left({d}_x,{\mathrm{WED}}_x\right)}}{{\mathrm{PDD}}_{\left({\mathrm{WED}}_{\mathrm{IVD}}\right)}\bullet{G}_{\left({d}_{\mathrm{IVD}},{\mathrm{WED}}_{\mathrm{IVD}}\right)}} \end{equation*}

where *D*_IVD_ represents the dose measured with the IVD detector. PDD_(WED)_ represents a percent-depth-dose calculated as a cubic approximation. The PDD was measured with a BluePhantom^2^ scanning water tank system (IBA Dosimetry) and the Farmer-type ionization chamber. The center of the water tank was placed at 400 cm from the radiation source, and the PDD was measured by moving the chamber horizontally. The gantry angle, collimator angle and the field size were the same to the setting of the patient treatment.

The WED at the evaluated point (WED*_x_*), the position of the proximal IVD detectors (WED_IVD, prox_), and the position of the distal IVD detectors (WED_IVD, distal_) from the surface of the acrylic wall of the TBI bed were calculated using following formulas:

(6) \begin{equation*} {\mathrm{WED}}_x={\mathrm{WED}}_{\mathrm{wall}}+{\mathrm{WED}}_{\mathrm{beads}}+{d}_{\mathrm{block}}+{\mathrm{EPL}}_x \end{equation*}

(7) \begin{equation*} {\mathrm{WED}}_{\mathrm{IVD},\mathrm{prox}}={\mathrm{WED}}_{\mathrm{mid}}-{\mathrm{EPL}}_{\mathrm{Mid}}+1.4\ \mathrm{cm} \end{equation*}

(8) \begin{equation*} {\mathrm{WED}}_{\mathrm{IVD},\mathrm{distal}}={\mathrm{WED}}_{\mathrm{mid}}+{\mathrm{EPL}}_{\mathrm{Mid}} \end{equation*}

where WED_wall_ represents the WED of the acrylic wall (1.18 cm) of the bed. For head and neck regions, this value was doubled to consider the acrylic plates beside the patient’s head. WED_beads_ represents the WED of the plastic beads. For head and neck regions, the air gap between the patient’s body and the acrylic plate was not considered for calculating WED. *d*_block_ represents the physical thickness of the solid water and the RW3 inserts used to compensate the head and neck doses. EPL*_x_* represents the EPL (equivalent path length) from the patient’s body surface to the evaluated point *x*, and the value is defined only inside the patient’s body. The EPL from the patient surface to the mid-plane (EPL_Mid_) was calculated using Eclipse TPS ver. 15.1 (Varian Medical Systems). To calculate EPL_Mid_, tentative beams were generated and reference points were placed at the mid-plane of the patient’s head, neck, chest and umbilicus levels. At the chest region, the EPL_Mid_ was evaluated for the patient’s body excluding the arms because the IVD detectors were attached to the patient’s body. The EPL*_x_* at arbitrary depth was calculated using following formula:

(9) \begin{equation*} {\mathrm{EPL}}_x={\mathrm{EPL}}_{\mathrm{Mid}}\bullet \frac{d_{\mathrm{x}}}{d_{\mathrm{Mid}}} \end{equation*}

Because the PDD was calculated using the WED, the values were corrected by the inverse square law, as shown in equation (5).

### End-to-end test and patient treatments

A RANDO phantom (The Phantom Laboratory, Salem, NY, USA) was used for end-to-end test. The computed tomography (CT) images of the phantom were acquired and imported to the Eclipse TPS. The physical and water-equivalent thicknesses were measured at the positions of the IVD measurements. The phantom was positioned on the bed and the gaps between the phantom body and the acrylic walls were filled with the plastic bead bags. The IVD detectors were attached, and the phantom was irradiated from both right–left and left–right directions by rotating the bed. The MU with the prescribed dose of 100 cGy for each beam was calculated using equation (1).

Following approval of the institutional review board, two patients who received RIST combined with TBI for treatment of malignant lymphoma (patient #1) and acute myeloid leukemia (patient #2) were retrospectively evaluated. The prescribed doses were 400 cGy/2 fractions/2 days and 300 cGy/1 fraction/1 day for patients #1 and #2, respectively. For these two patients, eye block and compensator for lung were not used because of the low prescribed dose. CT images were acquired in the supine position without beads a week before treatment to measure the physical and water-equivalent thicknesses (Supplementary Table 1, see online supplementary material). During the treatments, the patients were laid on a disposable sheet, and the plastic bead bags were filled between the sheet and the acrylic walls. First, the photon beam was irradiated from the patient’s right, and then the bed was rotated by 180° and the beam was irradiated from patient’s left. The IVD detectors were used to monitor the dose during treatments. Because the IVD detectors received beams from opposing directions, different calibration data were used for right–left and left–right beams. A Farmer-type ionization chamber was also placed between the patient’s thighs to measure the mid-plane dose. The chamber was covered with a plastic buildup cap and sandwiched with plastic bead bags.

### TPS calculation

**Fig. 2.**
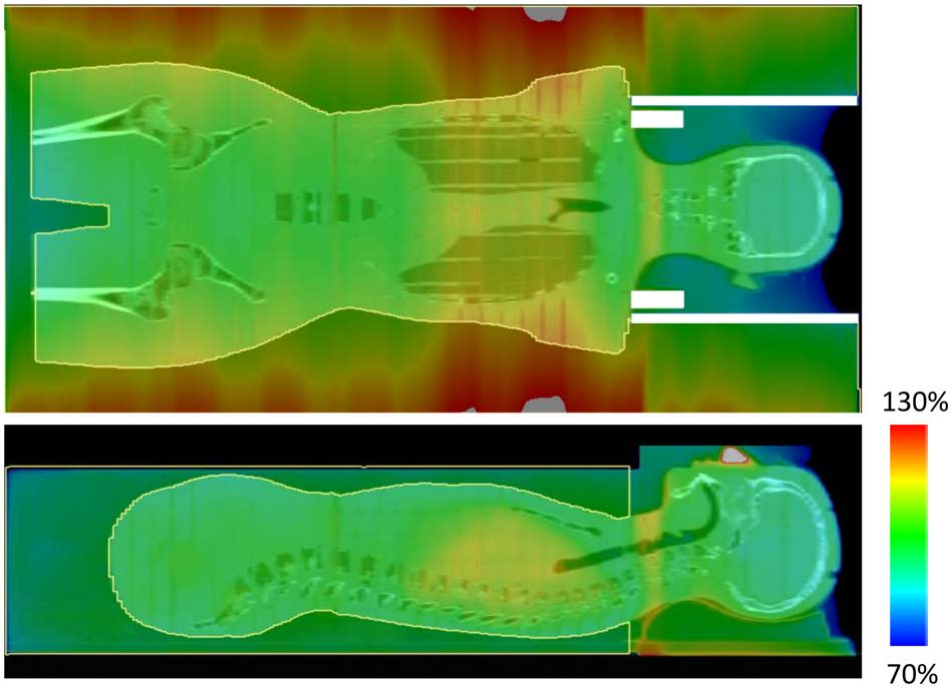
Dose distribution of the RANDO phantom calculated with the Acuros XB algorithm.

The dose calculation accuracy of the Eclipse TPS for long SSD was evaluated using CT images of the RANDO phantom and two patients (Fig. 2). The beam data of the TPS was modeled using the representative beam data (RBD) provided by the vendor. The structures of the acrylic walls, acrylic plates beside the head, 5-cm solid water phantoms and 2-cm RW3 blocks were virtually generated on the TPS. The materials of the solid water and RW3 were set as water, whereas those of the acrylic walls and plates were set as polymethylmethacrylate (PMMA). A cubic structure including whole body was generated to mimic the plastic bead bags. The height of the beads structure was determined by the height of the patient’s abdomen. The patient’s body, acrylic plates, RW3 blocks and the airgap around the head were subtracted from the beads structure. The CT value of the beads structure was −150 HU, which was calculated from the water-equivalent density of the plastic beads and the physical density–CT value conversion curve measured for modeling TPS. Two opposing fields were generated to simulate the irradiation. Two calculation algorithms were evaluated: an anisotropy analytical algorithm (AAA) ver. 15.1 and Acuros XB (AXB) ver. 15.1. The calculation grid sizes were 5 and 3 mm for AAA and AXB, respectively, which were the maximum grid sizes for these algorithms. For AXB, dose to medium was used. Whole body could not be calculated with AXB because of insufficient memory of the computer. Therefore, 4–6 copies of the treatment plan were generated for each patient and the dose calculations were performed for limited volumes. To generate the dose for whole body, the DICOM RT-Dose files of the plans were exported, and the voxel values of the DICOM file generated from the AAA plan were overwritten by those of the AXB plans. During CT image acquisition, towels were placed under the patient’s upper arms to overlap the arms with the lungs.

## RESULTS

**Fig. 3.**
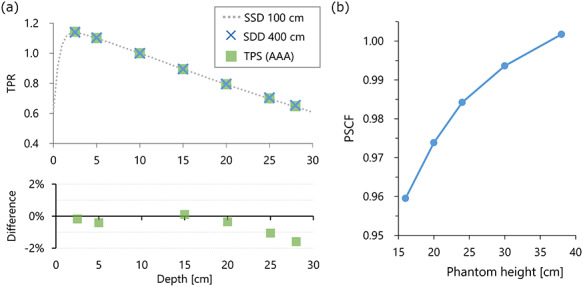
(**a**) TPR measured at 400 cm SDD. The TPR calculated using a TPS is also plotted. The line represents the TPR converted from the PDD measured with 100 cm SSD. The relative difference of the AAA from the values measured using the ionization chamber is plotted in the lower row. (**b**) PSCF plotted against the thickness of the phantom.

The values of DMU and SF were 0.0585 cGy/MU and 0.976, respectively. In Fig. 3a, cross points represent the TPR measured at an SDD of 400 cm. The line represents the TPR converted from the PDD measured at an SSD of 100 cm. The differences were small and within 1.6%. The TPR calculated using the Eclipse TPS was also plotted, showing similar results to the measured values. Figure 3b shows the PSCF plotted against the height of the phantom. The PSCF decreased with decreasing phantom height, and the value was 0.974 for 20 cm height.

**Fig. 4.**
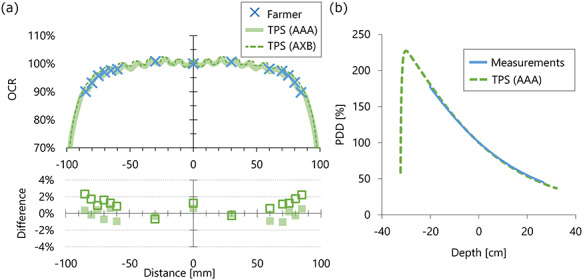
(**a**) OCR measured with a Farmer-type ionization chamber at 372 cm SSD and 28 cm depth at central axis. The values calculated using a TPS are also plotted. The differences of the TPS calculations from the values measured using the ionization chamber are plotted in the lower row. (**b**) The PDD measured with 367.7 cm SSD is plotted against the position from the center of the scanning phantom. The PDD calculated with the TPS with the same geometry is also plotted.

Figure 4a shows the OCR. The dose relative to the central axis was >90% in the range ± 80 cm from the central axis. Therefore, a dose of >90% is achievable if the patient’s whole body is included in the light field with 0° collimator angle and 40 × 40 cm^2^ field size. Because the OCR calculated using the Eclipse TPS showed the fluctuated profile, the values were normalized by the mean value in the range ± 10 cm. In the range ± 80 cm from the central axis, the differences between the measurements and TPS at measured points were within 1% and 1.8% for AAA and AXB, respectively. Figure 4b shows the PDD measured using the scanning water phantom. The PDD calculated using the Eclipse TPS was also plotted, showing differences from −1.9 to 4.1%. The TPS-calculated PDD showed a slightly steeper curve, indicating that the PDD measured with long SSD showed slightly higher photon energy.

**Fig. 5.**
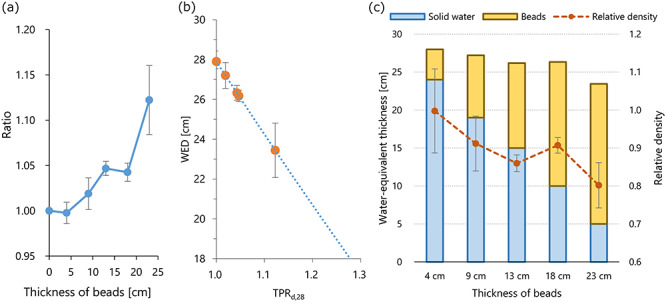
(**a**) The dose measured at 28 cm physical depth with replacing a part of the solid water with plastic beads. The doses relative to that measured in full solid water are plotted against the thickness of the beads. (**b**) Correlation between WED and the TPR relative to the value at 28 cm depth. The line represents the quadratic approximation function. Circles represent the WED for the ratio shown in (a) calculated with the approximation curve. (**c**) Columns represent the WEDs of the solid water and beads (left axis). Plotted points represent the density of the plastic beads relative to water (right axis).

To calculate the relative density of the plastic beads, the dose was measured at 28 cm depth by replacing part of the solid water with the plastic bead bags. Figure 5a shows the dose relative to that measured with full solid water, plotted against the thickness of the beads. The dose increased with the ratio of the beads. Figure 5b shows the correlation between the TPR_*d*, 28_ and the depth, and the line represents the quadratic approximation. Then the WED values were calculated for a combination of solid water and plastic beads. In Fig. 5c, columns represent the WED. The height of the ‘Beads’ represents the WED_solid water + beads_ minus *d*_solid_. The ρ_beads_ values are also plotted in Fig. 5c, and the mean ± SD was 0.90 ± 0.09.

To evaluate the accuracy of the point dose calculated with equation (2), the dose was measured in the I’mRT phantom with the same beam arrangement to the patient treatment. The differences from calculations were 0.11, −0.36 and − 2.32% for mid-plane, the position shifted by 3 cm and the phantom with Tough Lung, respectively. At the mid-plane, the difference from prescribed dose was −3.1%.

**Fig. 6.**
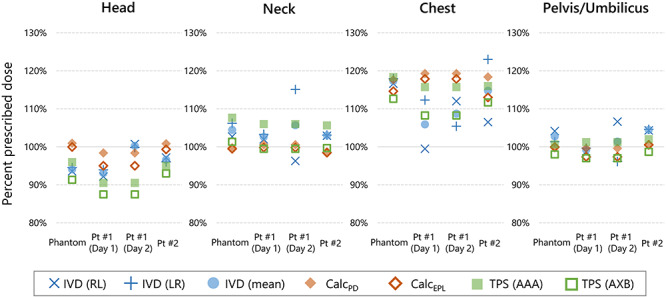
The percent prescribed dose at the mid-plane of the positions measured with IVD devices. The 100% prescribed dose of each lateral beam was 100, 100 and 150 cGy for the phantom, patient #1 and patient #2, respectively. Pt = Patient, RL = right-to-left, LR = left-to-right, Calc = manual calculation, PD = physical depth.

Figure 6 shows the mid-plane doses in the RANDO phantom and patients measured with IVD (*D*_IVD_), calculated manually with EPL (*D*_Calc, EPL_), calculated manually with physical depth (*D*_Calc, PD_) and calculated using the Eclipse (*D*_TPS, AAA_ and *D*_TPS, AXB_). Detail is shown in Supplementary Table 2 (see online supplementary material). Because compensator for lung was not used, the chest region received 110–120% of the prescribed dose. Except for the chest region, the mean *D*_IVD_ was within the range 93 to 106%. For the pelvis/umbilicus region, the mean *D*_IVD_ was within 5% of the prescribed dose. The mean ± SD (range) of the differences between *D*_Calc, EPL_ and *D*_Calc, PD_ was −1.5 ± 1.5% (−4.6 to 0.3%). For the RANDO phantom, the mean ± SD (range) of the difference between *D*_Calc, EPL_ and *D*_IVD_ was 1.1 ± 4.8% (−5.9 to 5.0%). For patients, the difference between *D*_Calc, EPL_ and *D*_IVD_ was 0.4 ±5.8% (−12.0 to 5.9%). Excepting the chest region, the differences were within ±6%. Although the manual calculation estimated an overdose in the lung region, the *D*_IVD_ was closer to the prescribed dose than expected, probably because the calculated dose was the worst case and the patient’s arms were not considered. The difference in the dose measured using an ionization chamber from the prescribed dose was 2.5 ± 5.2% (−2.2 to –9.6%).

**Fig. 7.**
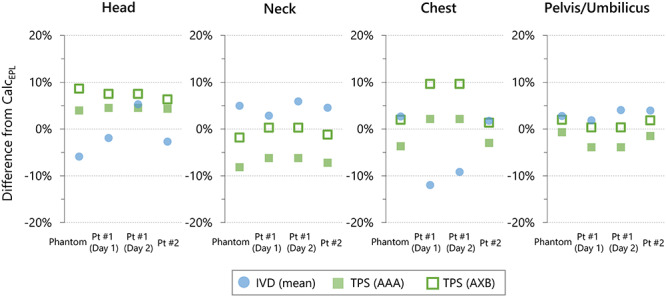
The Differences of the dose at the mid-plane of the positions measured with IVD devices and TPS calculations from the values manually calculated using the equivalent path length (Calc_EPL_). Pt = Patient, RL = right-to-left, LR = left-to-right.

For TPS calculations, the mean ± SD (range) of the differences between *D*_TPS, AXB_ and *D*_TPS, AAA_ were − 4.6 ± 1.5% (−6.5 to −2.1%). For the RANDO phantom, the differences between the TPS and the *D*_IVD_ were 1.0 ± 2.3% (−2.1 to 3.2%) for AAA and −3.8 ± 1.0% (−4.8 to −2.7%) for AXB (Fig. 7). For patient treatments, the differences were 0.8 ± 5.0% (−9.8 to 9.8%) for AAA and −4.0 ± 3.6% (−12.8 to 2.3%) for AXB. For patient #1 Day 2, the largest difference was observed at the head. As illustrated in Fig. 4a, the variations in the dose around the field edge is large. Although the central axis of the beam was set at patient umbilicus level, the patients were laid on the bed to include the whole body inside ±80 cm from the central axis. Patient position in the superior–inferior direction may be different during actual treatment.

**Fig. 8.**
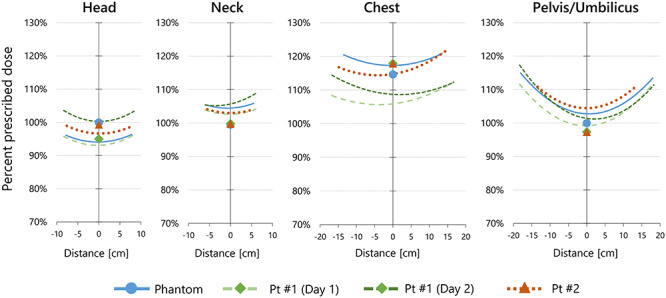
Depth–dose curve in the phantom or patients calculated from the dose measured with IVD detectors (lines). Points represent the mid-plane dose calculated using equivalent path length. Pt = Patient.

Figure 8 shows the depth–dose curve in phantom and patient body calculated with equation (5) and the dose measured with IVD. The point dose was calculated at positions from the proximal IVD to the distal IVD with a 1 mm interval. At each point, the mean of doses calculated with proximal and distal IVD doses was evaluated. The sum of the depth–dose curves for irradiation in the right–left and left–right directions are shown. *D*_Calc, EPL_ is also plotted as points. At the head and neck regions, uniformity ±10% was achieved for all patients at any depth. In the chest region, overdose >10% was observed because lung block was not used. At the mid-plane of the pelvis and umbilicus regions, dose uniformity ±5% was achieved for all patients. However, overdose >10% was observed around the patient’s body surface. This was because the width of the TBI bed including the acrylic walls is 58 cm. The energy of a 10 MV photon beam was not high enough to achieve uniform depth–dose for this thickness.

## DISCUSSION

In this study, we have performed the commissioning of the TBI using plastic bead bags and validated the method using phantoms. We also investigated the delivered dose using IVD detectors. Although the dose at the chest region showed overdose >10% from the prescribed doses because we did not use lung blocks, the plans were accepted by the physician because the prescribed doses were low. At the other regions, both manual calculations and mean of proximal and distal IVD doses achieved uniformity ±10% from the prescribed doses, indicating that commissioning of the method was appropriately performed. At the chest region, patient #1 Day 1 showed difference >10% between *D*_IVD_ and the *D*_Calc, EPL_. *D*_IVD_ includes various uncertainties such as variation of the patient posture, the detector position and direction. The detector positions were roughly determined by checking the sternal bone, but slight difference of the position might lead to the dose difference. For manual and TPS calculations, the doses were evaluated at the position where the patient’s arms were not on the beam path. However, the arms might be upstream of the detector during the treatments. The angular dependence of the IVD detectors has been reported [24], although it was difficult to know the direction of the detectors during the treatment.

The MU was calculated based on the measurements using a solid water phantom with 40 cm height. However, the actual height of the patient’s body is ~20 cm. The PSCF for 20 cm height was 0.974, indicating a decrease in the delivered dose of 2.6% from the condition of 40 cm height. The effective density of the plastic beads also affected the dose. The TPR used for MU calculation was measured with a 28 cm depth in the solid water. If the depth of 28 cm from the acrylic wall to the mid-plane includes 18 cm of the patient’s body and 10 cm of the plastic beads, the dose increases by ~4% because of shorter WED. Because these two effects compensated each other, the actual dose delivered to the patient’s mid-plane was close to the dose calculated based on the solid water. According to the vendor-provided data, attenuation of the plastic beads without the fabric bag against a 10 MV photon beams was 0.96–0.99 relative to that of solid water. However, the beads are encapsulated into fabric bags, whose density will be lower than that of the beads. As illustrated in Supplementary Figs 3 and 4, many bags are stacked between the acrylic wall and the patient’s body. The fabric bags and the air gaps between bags will result in a decrease in the effective density. The dose measured in the I’mRT phantom showed good agreement within 3% with the manually-calculated dose, indicating the validity of the effective density of the plastic beads evaluated in this study.

The dose measured with IVD detectors showed that the manually-calculated dose and the measured dose were within 5% of the prescribed dose at pelvis and umbilicus level, although the dose at the neck region for patient #1 Day 2 showed overdose >10%. After the treatment, the therapist noticed that the detector was not appropriately attached to the patient’s neck. The uncertainties of the dose at the neck region will be large because of various factors, including the body thickness where the detectors were attached, overlap of the RW3 insert, and patient shoulder position. For the chest region, the RANDO phantom showed good agreement between the manually-calculated and the IVD-measured doses. However, the IVD-measured doses during patient treatments were lower than the manual calculations. This is because the patient’s arms were not considered for the calculation of the PD and EPL. Although the worst case was evaluated by manual calculations, attenuation in the patient’s upper arms and humerus would improve the dose uniformity.

The Japanese Radiation Oncology Study Group (JROSG) recently reported the national survey on TBI prior to RIST [6]. They reported that 57.7% of institutions used the prescribed dose of 4 Gy/2 fractions/1–2 days. Shielding of lungs and lenses were performed at 43.6 and 50.0% of institutions, respectively. JROSG also reported the national survey for myeloablative TBI and reported that ~80% of institutions routinely shielded lungs [4]. For myeloablative treatments, management of lung dose is important. According to the survey, 61 and 31.7% of institutions used a dose rate of 10–15 cGy/min and <10 cGy/min, respectively. Gao *et al*. previously reported that a dose rate ≥15 cGy/min significantly increased the risk of post-transplantation idiopathic pneumonia syndrome [25]. In our institution, the DMU, SF and TPR_28,10_ were 0.0585 cGy/MU, 0.976 and 0.654, respectively. Therefore, the dose rate at 28 cm WED was 7.47 cGy/min. A dose rate <10 cGy/min will be achievable even if the dose rate in lungs increases because of low density.

We also evaluated the calculation accuracy of the Eclipse TPS. For the RANDO phantom, the TPS calculation showed good agreement with the IVD-measured dose within 5%. For patients, the AAA calculation showed agreement within 10%. The OCR calculated with the TPS showed fluctuating curves. The PDD also showed slight difference in the photon energy. Usually commercial TPSs are used for isocentric treatments for photon beam therapy, and they may not be considered for use for long-SSD. Lamichhane *et al*. previously evaluated the calculation accuracy of the AAA and AXB under long-SSD conditions [26]. They also reported that the TPS-calculated PDD was lower than measured PDD, and the accuracy of the point dose calculation in solid water was overestimated by up to 4.9%. Although our data also showed the inaccuracy of the Eclipse TPS for long-SSD calculations, it may be used to roughly estimate patient dose.

The AAPM task group 29 (TG-29) recommended that the minimum phantom size is 30 × 30 × 30 cm^3^ [11]. In this study, the DMU was determined using a 40 × 40 × 40 cm^3^ solid water phantom. However, limited phantom scatter was not considered. AAPM TG-29 provided a table of the correction factors for limited phantom scatter. However, we also have to consider that the height of the patient’s body is thinner than solid water. In this study, we considered the PSCF for calculations of the mid-plane doses and showed good agreement within 3% with dose measured in the I’mRT phantom. We consider that the accuracy of the procedure established in this study was good enough for clinical applications. However, the uncertainties related to patient setup affect the dose delivered to patients. In particular, patient shoulder position during CT image acquisitions affected the position of acrylic plates and RW3 inserts. If the condition of patient knee-bending changes, the patient position on the bed will also change, resulting in dose uncertainty at the patient’s head. For accurate dose estimation, careful attention to patient setup will be needed.

We have established the technique of TBI using plastic bead bags. This methodology will be useful for institutions that do not have experience of such techniques. For myeloablative conditioning, further investigations for lung and lens shielding will be needed. However, the shielding methodology will be the same as that used with standard long-SSD treatments. Although we do not recommend the use of TPS for the determination of MU, it may be useful to roughly estimate patient dose.

## Supplementary Material

Suppl_Figure_1_rraa072

Suppl_Figure_2_rraa072

Suppl_Figure_3_rraa072

Suppl_Figure_4_rraa072

Suppl_Table_R1_rraa072
