# Supplementary figures and images for: Commissioning of total body irradiation using plastic bead bags
Source: J Radiat Res. 2020 Sep 2;61(6):959–68. doi: 10.1093/jrr/rraa072 (PMC7674696; doi:10.1093/jrr/rraa072)

(a)

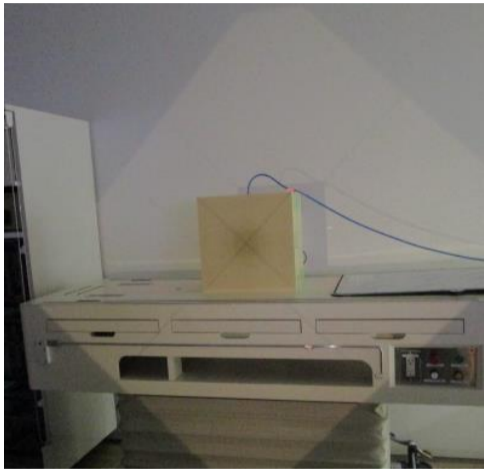

(b)

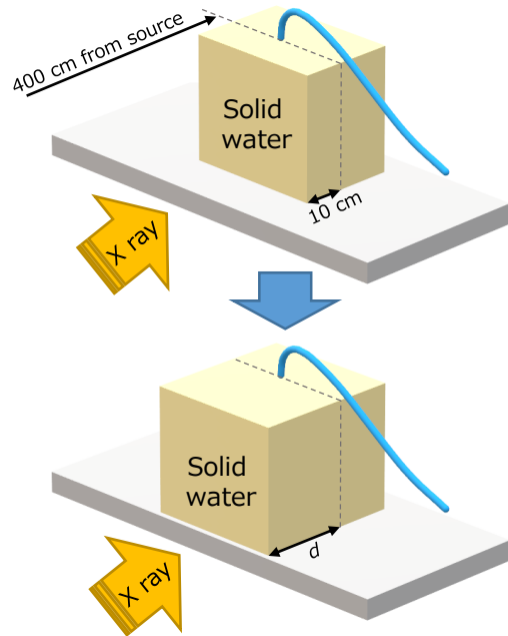

Supplement: Suppl_Figure_1_rraa072 [file suppl_figure_1_rraa072.pdf]

(a)

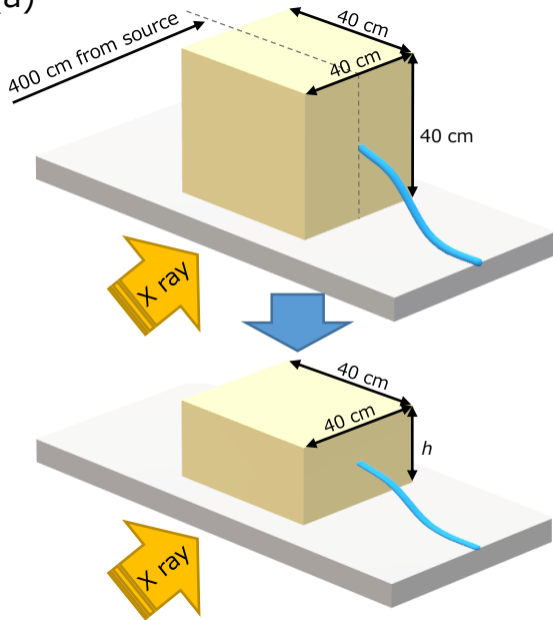

(b)

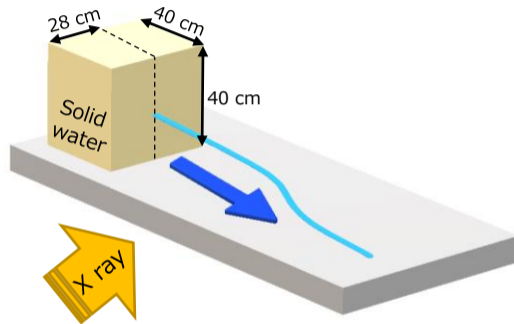

Supplement: Suppl_Figure_2_rraa072 [file suppl_figure_2_rraa072.pdf]

(a)

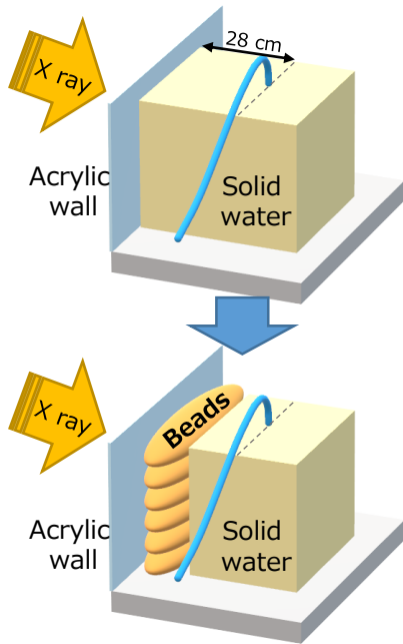

(b)

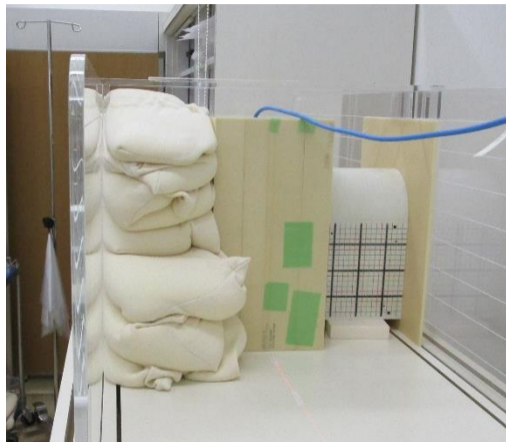

Supplement: Suppl_Figure_3_rraa072 [file suppl_figure_3_rraa072.pdf]

(a)

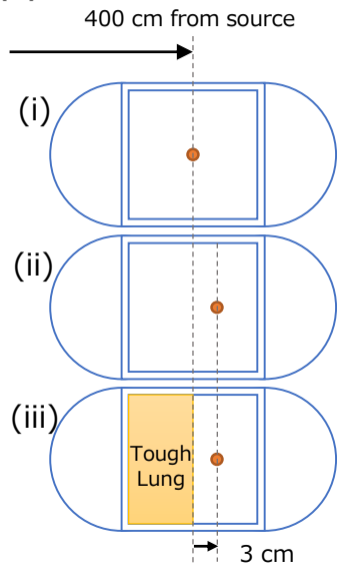

(b)

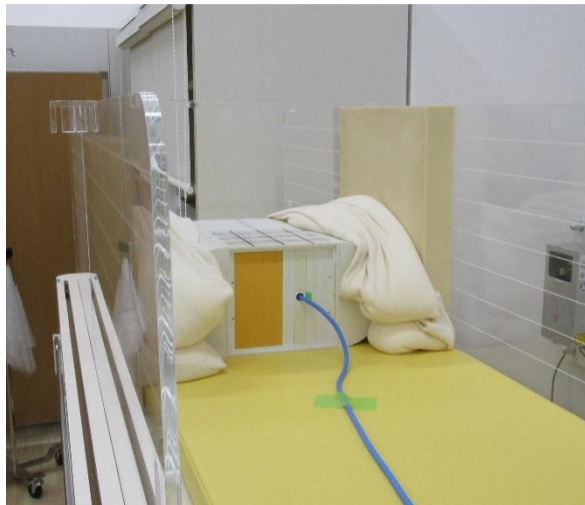

Supplement: Suppl_Figure_4_rraa072 [file suppl_figure_4_rraa072.pdf]
