# Supplementary material for: Commissioning of total body irradiation using plastic bead bags
Source: J Radiat Res. 2020 Sep 2;61(6):959–68. doi: 10.1093/jrr/rraa072 (PMC7674696; doi:10.1093/jrr/rraa072)
Supplement: Suppl_Table_R1_rraa072 [file suppl_table_r1_rraa072.docx]

Supplementary Table 1. Physical and water-equivalent depths from body surface to mid-plane at IVD measurement points.

| Patient | Lung |  | Head | Neck | Chest (corr) | Pelvis/Umb |
| --- | --- | --- | --- | --- | --- | --- |
| Phantom | 9 cm | PD [cm] | 7.8 | 5.5 | 13.5 (7.2) | 18.0 |
|  |  | EPL [cm] | 8.2 | 5.6 | 8.3 | 17.9 |
|  |  |  |  |  |  |  |
| Patient #1 | 10 cm | PD [cm] | 8.4 | 6.0 | 16.7 (9.7) | 18.3 |
|  |  | EPL [cm] | 9.7 | 6.3 | 10.2 | 19.2 |
|  |  |  |  |  |  |  |
| Patient #2 | 9.6 cm | PD [cm] | 7.6 | 5.5 | 14.9 (8.1) | 13.4 |
|  |  | EPL [cm] | 8.2 | 5.6 | 10.1 | 13.4 |

Abbreviations: Umb, umbilicus; PD, physical depth; EPL, equivalent path length; corr, the physical depth at chest region subtracted by the thickness of the lung multiplied by 0.7.

Note: Dose at greater trochanter (pelvis) level was measured for the phantom and patient #1, whereas the dose at umbilicus level was measured for patient #2.

Supplementary Table 2. The absolute doses (cGy) measured using the in vivo dosimetry (IVD) detectors and calculated using the Eclipse treatment planning system (TPS).

| Region | Parameter | Phantom | Pt #1  Day 1 | Pt #1  Day 2 | Pt #2 |
| --- | --- | --- | --- | --- | --- |
|  |  |  |  |  |  |
| Head | IVD (mean) | 94.1 | 93.1 | 100.3 | 144.9 |
|  | Calc (PD) | 101.0 | 98.4 | 98.4 | 151.3 |
|  | Calc (EPL) | 100.0 | 95.0 | 95.0 | 149.0 |
|  | TPS (AAA) | 96.0 | 90.5 | 90.5 | 142.5 |
|  | TPS (AXB) | 91.3 | 87.5 | 87.5 | 139.5 |
|  |  |  |  |  |  |
| Neck | IVD (mean) | 104.5 | 102.6 | 105.7 | 154.5 |
|  | Calc (PD) | 99.7 | 100.6 | 100.6 | 148.1 |
|  | Calc (EPL) | 99.5 | 99.8 | 99.8 | 147.7 |
|  | TPS (AAA) | 107.7 | 106.0 | 106.0 | 158.5 |
|  | TPS (AXB) | 101.3 | 99.5 | 99.5 | 149.5 |
|  |  |  |  |  |  |
| Chest | IVD (mean) | 117.3 | 105.9 | 108.7 | 172.1 |
|  | Calc (PD) | 117.7 | 119.3 | 119.3 | 177.6 |
|  | Calc (EPL) | 114.6 | 117.9 | 117.9 | 169.5 |
|  | TPS (AAA) | 118.3 | 115.8 | 115.8 | 174.0 |
|  | TPS (AXB) | 112.7 | 108.3 | 108.3 | 167.5 |
|  |  |  |  |  |  |
| Umb | IVD (mean) | 102.8 | 99.2 | 101.4 | 156.7 |
|  | Calc (PD) | 99.7 | 99.6 | 99.6 | 150.8 |
|  | Calc (EPL) | 100.0 | 97.3 | 97.3 | 150.8 |
|  | TPS (AAA) | 100.7 | 101.3 | 101.3 | 153.0 |
|  | TPS (AXB) | 98.0 | 97.0 | 97.0 | 148.0 |
|  |  |  |  |  |  |
| Prescription (cGy/day) | | 100 | 100 | 100 | 150 |

Abbreviations: Umb, pelvis and umbilicus; Calc, manual calculation; PD, physical depth; EPL, equivalent path length; AAA, Anisotropy Analytical Algorithm; AXB, Acuros XB.

Note: Dose at greater trochanter (pelvis) level was measured for the phantom and patient #1, whereas the dose at umbilicus level was measured for patient #2.
